# The Morphometrics of “Masculinity” in Human Faces

**DOI:** 10.1371/journal.pone.0118374

**Published:** 2015-02-11

**Authors:** Philipp Mitteroecker, Sonja Windhager, Gerd B. Müller, Katrin Schaefer

**Affiliations:** 1 Department of Theoretical Biology, University of Vienna, Althanstrasse 14, 1090 Vienna, Austria; 2 Department of Anthropology, University of Vienna, Althanstrasse 14, 1090 Vienna, Austria; University of Naples, ITALY

## Abstract

In studies of social inference and human mate preference, a wide but inconsistent array of tools for computing facial masculinity has been devised. Several of these approaches implicitly assumed that the individual expression of sexually dimorphic shape features, which we refer to as maleness, resembles facial shape features perceived as masculine. We outline a morphometric strategy for estimating separately the face shape patterns that underlie perceived masculinity and maleness, and for computing individual scores for these shape patterns. We further show how faces with different degrees of masculinity or maleness can be constructed in a geometric morphometric framework. In an application of these methods to a set of human facial photographs, we found that shape features typically perceived as masculine are wide faces with a wide inter-orbital distance, a wide nose, thin lips, and a large and massive lower face. The individual expressions of this combination of shape features—the masculinity shape scores—were the best predictor of rated masculinity among the compared methods (*r* = 0.5). The shape features perceived as masculine only partly resembled the average face shape difference between males and females (sexual dimorphism). Discriminant functions and Procrustes distances to the female mean shape were poor predictors of perceived masculinity.

## Introduction

Many studies have investigated whether the phenotypic “masculinity” of men, especially in facial appearance, plays a role in human mate preference and social perception (e.g., [1–3]). In these contexts, masculinity is assumed to be determined by the expression of steroid hormones, such as testosterone, in development. Since these hormones are immunosuppressive, a masculine phenotype is considered an honest signal of immunocompetence and mate quality (e.g., [4]). Yet, empirical evidence for the association between phenotypic facial masculinity, perceived masculinity, and attractiveness in humans remains scarce and inconsistent [5–10]. Body height has been found to play an important role in studies of social inference and mate choice [11,12]. Hence, facial allometry—aspects of face shape reflecting body size—is likely to contribute to the perception of facial masculinity [13].

Early attempts to construct “masculine faces” for rating studies were mainly based on manual image manipulation, without the use of empirical morphometric data (e.g., [14–16]). Other studies, based on simple morphometric approaches (using linear distances and distance ratios), intended to provide a quantitative measure of masculinity (a “masculinity score”) for actually measured faces (e.g., [17,18]). Modern morphometrics and image analysis have opened an array of new possibilities for multivariate studies of the perception of human faces and bodies (e.g., [19,20–22]). Among these, geometric morphometrics allows for a powerful visualization of shape differences and for the construction of artificial faces based on statistical estimates. The wide range of methods that have been used to generate continuous masculinity scores from morphometric variables differ considerably in their statistical and biometric properties. In many of these approaches, the underlying biological and psychological concepts of masculinity were not properly distinguished and the statistical techniques remained unjustified (see below).

In this paper we outline a strategy for estimating biologically and statistically meaningful notions of masculinity. We then critically review other morphometric approaches to masculinity in the current literature and discuss possible biological and psychological interpretations (see Table 1 for a summary). Finally, we demonstrate these methods by an application to a dataset of human facial photographs—still the most common data source in face research [23].

**Table 1:** A selection of different masculinity concepts and their statistical properties.

Masculinity concept	Reference data	Computation	Interpretation
Perceived masculinity(e.g., [22,29])	Rating by naïve subjects	Multivariate regression of morphometric variables on the masculinity rating	Morphological pattern driving the masculinity rating
Hormone-mediated masculinity(e.g., [22])	Measurement of sex steroid levels (postnatally: salivary, blood; prenatally: approximated by 2D:4D)	Multivariate regression of morphometric variables on hormone level or 2D:4D	Morphological effect of the measured hormone
Average morphological sexual dimorphism (e.g., [3,19,53,54,])	Average male and average female morphology	Difference between male and female mean shapes	Average morphological effect of sex chromosomes (XY, XX)
Allometric versus non-allometric sexual dimorphism (e.g., [32, 38])	Average male and average female morphology; a measure of size of the investigated structures	Regression of morphology on both size and sex	Sexual dimorphism in shape resulting from dimorphism in size versus size-independent dimorphism
Sum of standardized dimorphic traits(e.g., [17,18,42])	Prior selection of dimorphic traits; standard deviation of each variable	Sum of standardized measurements	Variables with low sexual dimorphism have high weights; no obvious multivariate biometric interpretation
Linear discriminant function(e.g., [7,20])	Average male and average female morphology; within-sex covariance matrix	Mean difference vector multiplied by the inverse within-sex covariance matrix	Classification technique based on dimorphic variables with low variance within the sexes
Deviation from female mean shape(e.g., [43])	Average female morphology	Masculinity scores are given by the Procrustes distance between each shape and the female mean shape; no corresponding axis in shape space	Deviation from female mean shape in all directions of shape space, including non-dimorphic features

### The morphometrics of masculinity

Chromosomal sex is a binary property. Except for chromosomal aberrations, a person is either male (XY) or female (XX). “Masculinity”, by contrast, is a continuous property that refers to two different concepts. First, it can designate the individual variation of shape features that differ on average between the sexes. Such sexually dimorphic features are commonly referred to as secondary sex characteristics (such as body height, skeletal structure, muscle mass, facial and body hair, mandibular prominence), which typically are mediated by sex steroids. In this paper we use the term *maleness* (or *femaleness*) in the sense of a continuous score that reflects the individual expression of sexually dimorphic features. Second, the term masculinity can designate *perceived masculinity*—a graded property ascribed by one or several observers.

Whereas maleness is a purely morphological concept, perceived masculinity is a psychological concept—a mental construct—that is typically assessed via rating studies. It is an empirical question to which degree perceived masculinity resembles maleness. Multiple studies reported that variation in human facial secondary sex characteristics correlates with ratings such as masculinity/femininity, dominance, and attractiveness, as well as with estimates of hormone status (e.g., [19,24–26]). Cross-cultural agreement for trait attributions to faces is high and even generalizes to face-like inanimate objects (e.g., [27]).

Morphometric studies of facial masculinity thus need to distinguish between (a) average sexual dimorphism in face shape, (b) individual variation of sexually dimorphic shape features within the sexes, and (c) shape features affecting perceived masculinity.

The morphological pattern that drives, on average, a psychological rating such as perceived masculinity, can be estimated by a multivariate regression of morphological traits on the rating scores (e.g., [22,28,29]). Suppose we have *p* morphometric measurements *x*_1_, *x*_2_, …, *x*_*p*_ of *n* human faces. Regressing the morphometric variables (e.g., Procrustes shape coordinates) on a masculinity rating results in a vector of regression slopes that comprises the *p* univariate regression slopes *b*_1_, *b*_2_, …, *b*_*p*_. One can compute faces with different perceived masculinity by adding corresponding multiples of the vector to a reference configuration. In geometric morphometrics and several image analysis approaches, these estimated morphological configurations can be displayed as actual shapes or images [21,28,30]. The vector of regression slopes also serves as an axis in the *p*-dimensional data space, and the *n* coordinates or scores of the assessed individuals along this axis can be computed as the linear combination (weighted sum) *b*_1_ *x*_1_ + *b*_2_ *x*_2_ + … + *b*_*p*_ *x*_*p*_. When standardizing the vector of regression coefficients to unit length, the scores can be interpreted as the orthogonal projections of the data points onto the vector (regression scores). We refer to these scores as *masculinity shape scores*.

The vector of multivariate regression slopes can be interpreted as a linear gradient in shape space that contrasts shapes with low versus or high masculinity rating. The scores along this gradient are a linear combination of the shape variables, weighted by their covariance with the rating. This linear combination of morphometric variables thus has the maximum possible covariance with the rating (it can be considered a two-block partial least squares analysis where one block consists of a single variable only). If the rating lacks a proper scale, the vector of multivariate regression slopes may be substituted by the difference vector between average shapes of high-rated and low-rated individuals. In contrast to a multivariate regression, partial regression coefficients from a multiple regression of the rating on morphology are more difficult to interpret and often computationally unstable (see below).

Multivariate regression can also be used to estimate the morphological pattern induced by the expression of androgenic hormones, such as testosterone (e.g., [5,26]), but a meaningful quantification of hormone levels is difficult. Testosterone concentration in adults may not be a reliable indicator of hormone activity during development. Other authors instead used the 2D:4D ratio (the length of the index finger relative to the length of the ring finger) as a proxy of prenatal testosterone exposure and based shape regressions on this variable (e.g., [19,26,31]).

**Fig 1:**
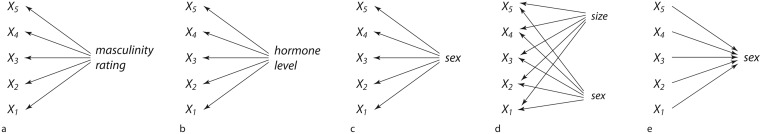
Path models corresponding to different multivariate methods of estimating masculinity. (a) The morphological pattern underlying perceived masculinity can be estimated by a multivariate regression of the morphometric variables (*X*_1_…*X*_5_) on a masculinity rating. (b) The morphological effects of steroid hormones can be estimated by a multivariate regression of the morphometric variables on a measure of hormone level. (c) The difference between average male and average female shape is equivalent to a regression of morphology on sex (as a binary variable). (d) Allometric and non-allometric components of sexual dimorphism can be estimated by regressing morphology on both size and sex. (e) A discriminant function is computationally equivalent to a multiple regression of sex on the morphological measurements.

**Fig 2:**
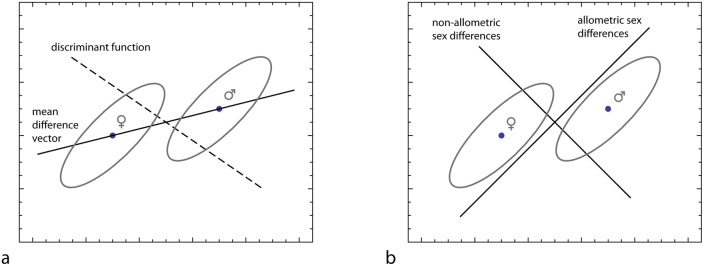
The statistical distribution of two morphometric variables for two groups of individuals (males and females) is shown by two equal frequency ellipses and the corresponding means. (a) The mean difference vector (solid line) is spanned by the two mean configurations. The discriminant function (dashed line) maximizes the squared distance between the group means relative to the variation of the scores within the groups. When the two covariance matrices are the same (as in this example), it is the optimal direction to discriminate the two groups and to classify individuals with unknown group membership. (b) The mean difference vector can be decomposed into an allometric component (which, for many morphometric data sets, is close to the direction of maximum variance within the groups) and a non-allometric component (orthogonal to the allometric direction).

The average difference between male and female shapes, which can be considered a regression of morphology on chromosomal sex (Fig. 1), is a vector comprising the *p* mean differences for all variables. It is an axis in the *p*-dimensional data space (Fig. 2a) along which scores for the assessed individuals can be computed. These scores are a linear combination of the original variables with weightings equal to the mean difference for each variable (scaled to unit sum of squares). Thus we obtain the linear combination with maximum sexual dimorphism. We refer to these continuous scores as *maleness shape scores* in order to distinguish them from masculinity shape scores. The maleness shape scores are morphometric estimates of maleness (individual expression of dimorphic features), whereas masculinity shape scores are morphometric estimates of perceived masculinity. Komori et al. called the difference vector between male and female mean shapes the “sex-relevant vector” and the subspace perpendicular to this vector the “sex-irrelevant vectors” [32].

Taller persons tend to have different body proportions (such as longer limbs relative to the head) and often also different facial proportions as compared with shorter persons (e.g., larger faces relative to the braincase). These relationships are similar across human populations [33]. Since, on average, men are taller than women, some of the average sex differences in body shape and face shape may owe to these differences in stature (induced mainly by differences in the expression of growth hormone and other growth factors) and not to the differential effects of androgen hormones. In biology, the association between the size and the shape of a body, or of body parts, is referred to as allometry (e.g., [34,35]). It is a classic approach in morphometrics to decompose a group difference into an allometric part and a non-allometric part [35–38]. Allometry can be estimated by regressing the shape variables on a measure of overall size within adults (static allometry) or within an ontogenetic sample (ontogenetic allometry). For studying facial allometry, typical size measures are body height and some estimate of facial size (e.g., centroid size). While body height and facial size are highly correlated throughout ontogeny, the choice of a size measure can be crucial in samples of adult individuals [13]. In the empirical analysis below, we use body height as a size measure because of its potential role in trait attribution.

The non-allometric part of sexual dimorphism is taken as the part orthogonal to allometry in the data space or, alternatively, as the sex mean difference in the residuals from the regression on size [39]. Schaefer et al., for instance, found that the ratio of allometric to non-allometric sexual dimorphism correlates with the relative importance of male-male competition versus female choice and sperm competition in the social structure of higher primates [38]. Non-allometric sexual dimorphism thus may more closely resemble the effects of androgens than the allometric part.

### Further published approaches to the measurement of masculinity

Brown et al. [20] and Scott et al. [7] proposed a linear discriminant function between males and females to represent “an objective measure of masculinity” ([7], page 8). Under the (often unrealistic) assumption of homogenous variance-covariance matrices, a discriminant function is a tool for maximum likelihood classification. The linear discriminant function between two sexes is the vector for which the squared difference between male and female average scores is a maximum *relative* to the variation of the scores within the sexes (e.g., [40,41]). Hence, sexually dimorphic traits with large variance within the sexes contribute less to the discriminant function than variables with low within-sex variance. It is not *a priori* clear that such a score resembles the human perception of masculinity. Computationally, the discriminant function is equal to a regression of sex (as a binary variable) on morphology, which conveys no obvious biological meaning (Fig. 1e; [41]). A considerable problem can be the dependence of the discriminant vector on the exact list of variables. Adding or skipping a variable may modify the coefficients of all other variables. In many morphometric applications, the data are first reduced to a small number of principal components (PCs) before computing a discriminant function, because a stable computation requires many more individuals than variables [41]. But the discriminant function typically depends on the number of selected PCs. Brown et al. [20] and Scott et al. [7] performed a step-wise elimination of principal components in the course of their discriminant function analysis. But this does not circumvent the problem, and, unlike other variables, principal components cannot be arbitrarily selected because of their hierarchical way of computation.

In other studies, the sum of a small selection of supposedly androgen-responsive traits was used for a measure of masculinity. Scheib et al., for example, computed a masculinity index by standardizing (z-transforming) and summing relative lower face length and relative cheekbone width [17]. Penton-Voak et al. [18] and Burriss et al. [42] derived a masculinity index by the summing of several standardized traits (distance measurements) that had significantly higher values in men and subtracting from that sum the traits that had higher values in women. Such indices are linear combinations of the original variables with weights equal to plus or minus the standard deviation of the respective variable. Since the standard deviation comprises both variability within the sexes and between the sexes (i.e., sexual dimorphism), variables with small sexual dimorphism contribute more to this kind of masculinity score than highly dimorphic variables. This is probably not what most researchers intend. The summing of untransformed variables would give an unweighted linear combination to which all selected variables contribute equally, regardless of their actual contribution to sexual dimorphism or some measure of masculinity. Both variants of this approach cannot be used in geometric morphometrics and other multivariate contexts.

Sanchez-Pages and Turiegano suggested using Procrustes distance between the shape of a male individual and the average female shape as a measure of masculinity [43]. Procrustes distance is a measure of overall shape difference, usually approximated by the Euclidean distance between the two superimposed configurations of landmarks (measurement points). An interpretation of this measure as a masculinity score is problematic because it summarizes deviations from average female shape in all directions of shape space, including deviations along the average male-female axis, but also along the directions perpendicular to it (corresponding to shape features with identical averages in both sexes). Furthermore, because Procrustes distance always is positive, males with a more feminine shape than the female average would still have a positive masculinity score.

For a comparison of these methods, we applied them to a small sample of human facial photographs, the most common data source in face research despite the limitations imposed by the two-dimensional representation. We estimated perceived masculinity and sexual dimorphism (maleness), which we decomposed into an allometric and a non-allometric part. Furthermore, we computed a discriminant function and the Procrustes distances between the male shapes and the female average in order to demonstrate the problematic behavior of these two approaches.

## Empirical Analysis

### Material & Methods

Our sample comprises frontal photographs of 21 Caucasian women (age 20–34 years) and 24 men (age 20–33 years) from the Viennese student population, who were recruited on campus. A camera with 200 mm lens was positioned at the eye height, 3.5 m away from the face. The heads were adjusted according to the Frankfort Horizontal Plane, and a scale bar was placed next to each head. In addition, body height and body weight were measured for each individual. Participation in the study was voluntary and based on written consent. Each participant was informed about the project and the measurement procedure.

For the rating, the male facial photographs were standardized with regard to white balance, contrast, and brightness. The images where then transformed into grey scale and superimposed by a blurred ellipse to disguise contextual information such as hairstyle and clothing. The final stimulus, including the face, the ellipse, and the uniform grey background, was the same size for all faces in order not to provide any direct size cues. Ninety-one age-matched (24.36 ± 3.49 years) female, self-reported heterosexual Caucasian subjects participated in the rating of the male faces. The raters were students and approached at the university of Vienna. No rater reported to have seen any photographed man before. They were asked to judge masculinity using a slider, ranging from “feminine” to “masculine”. The scale was hidden for the participants and consisted of a continuous range from 0 to 80. Only one face was presented at a time in a pseudo-randomized order. There was a minimum of 17 raters for each face.

**Fig 3:**
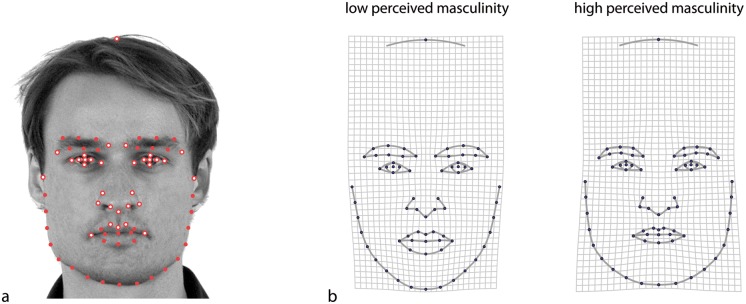
Landmark configuration used for studying face shape and perceived masculinity. (a) Face with the 33 landmarks (open circles) and 37 semilandmarks (filled circles) used in the morphometric analysis. (b) The shape features determining perceived masculinity are visualized by deformation grids from the mean shape to shapes predicted for deviations of ±20 rating scores from the average.

For the morphometric analysis we digitized 33 anatomical landmarks and 37 semilandmarks on each original image to describe overall facial form (Fig. 3A; see [29] for details). Semilandmarks are points on curves, for which the exact location along the curve cannot be identified and hence is statistically estimated. We used the sliding landmark algorithm for this purpose, which minimizes the bending energy (a measure of local shape difference) between each individual and the sample average [44,45]. After sliding the semilandmarks, the 45 landmark configurations were symmetrized by averaging each configuration with its relabeled reflection [21,46]. Subsequently the landmarks were superimposed by Generalized Procrustes Analysis [13,47].

The face shape patterns relating to perceived masculinity and allometry were calculated by multivariate regressions of the male face shapes on the masculinity rating and body height, respectively. Sexual dimorphism was computed as the difference between average male and average female shape. The non-allometric component of sexual dimorphism was computed as the difference between average male and average female shape after projecting out the allometry vector (projection of the sexual dimorphism vector in the subspace perpendicular to the allometry vector; [37,48]). All shape patterns were visualized using thin plate spline deformation grids [28,49].

### Results

The regression of facial shape on rated masculinity indicated that male faces with a higher masculinity attribution tended to have wider faces with a wider inter-orbital distance, a wider nose, thinner lips, and a larger, more rounded lower facial outline (Fig. 3B). This shape pattern, as a vector in shape space, accounted for 19.4% of total facial shape variation in males, and the corresponding masculinity shape scores had a Pearson product-moment correlation with the masculinity rating of 0.50.

**Fig 4:**
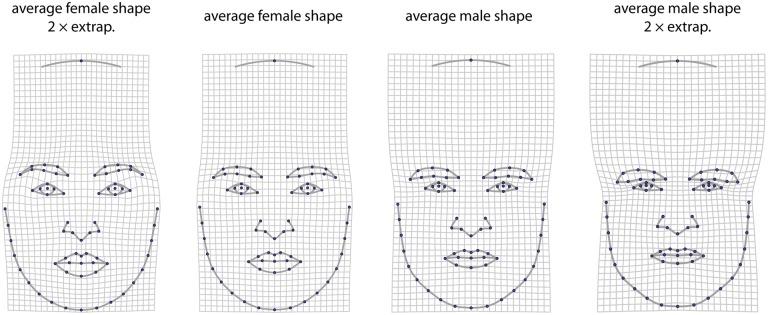
Sexual dimorphism is visualized by deformation grids between average female and average male facial shape, together with two-fold extrapolations of these shape differences.

Fig. 4 contrasts female and male mean shapes. Men, on average, had thicker and lower positioned eyebrows, relatively smaller eyes, thinner lips, and a more massive and angulated lower jaw than women. These dimorphic shape features, as a vector in shape space, accounted for 15.4% of total variation across all male face shapes, and the individual maleness shape scores along this vector had a correlation with the masculinity rating of 0.26.

**Fig 5:**
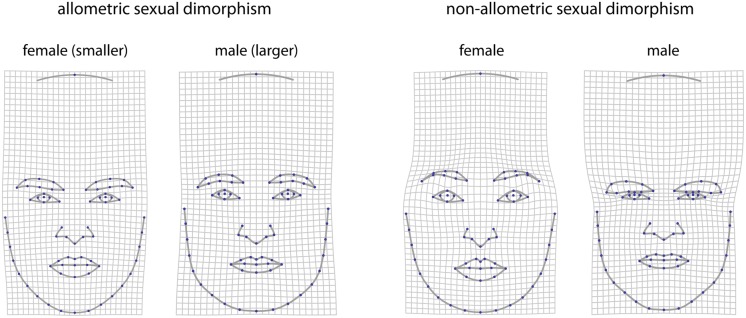
Decomposition of sexual dimorphism into an allometric and a non-allometric component. The corresponding deformation grids are two-fold extrapolations of the actual dimorphism.

Fig. 5 shows a decomposition of sexual dimorphism into an allometric (size-dependent) and a non-allometric (size-independent) component. Larger faces tended to have relatively wider and more angulated jaws along with relatively thinner lips as compared to smaller faces. Whereas the allometric component was very similar to the shape pattern underlying perceived masculinity (Fig. 3B), the non-allometric component more closely resembled the original sexual dimorphism (Fig. 4). As vectors in shape space, sexual dimorphism had an angle of 73° with the allometric component and of 17° with the non-allometric component. The individual shape scores along the allometric component were more variable within males than the scores along the non-allometric component (18.2% and 11.4% of total shape variation). The allometric shape scores were also more strongly correlated with rated masculinity than were the non-allometric scores (0.34 and 0.19, respectively). The resemblance of allometric shape (Fig. 5 left) and the shape pattern of perceived masculinity (Fig. 3B) results from the correlation of 0.43 between facial size (centroid size of the facial landmarks) and rated masculinity (note that all faces were scaled to the same size for the rating). When regressing both the shape coordinates and the natural logarithm of centroid size on perceived masculinity, the *form scores* along the resulting vector in form space (containing information on both shape and size) had a correlation of 0.51 with the masculinity rating (for more details on Procrustes form space see [13,37]).

**Fig 6:**
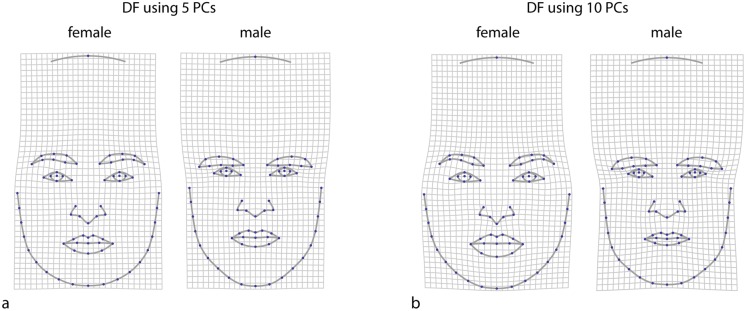
Visualization of discriminant functions between male and female face shapes using (a) five and (b) ten principal components (PCs) of the full set of shape coordinates.

Following Brown et al. [20] and Scott et al. [7], we also computed a linear discriminant function between males and females based on the first 5 principal components (accounting for 74% of total shape variation) as well as on the first 10 principal components (91% of total shape variation). The two discriminant functions to some degree resembled sexual dimorphism (Fig. 4) but differed in their combination of dimorphic traits (Fig. 6). Both sets of discriminant scores had a very low correlation with the masculinity rating of 0.03 and 0.14, respectively. In addition, we computed Procrustes distances between the male face shapes and the average female face shape as suggested by Sanchez-Pages and Turiegano [43]. These distances had a correlation with the masculinity rating of 0.19.

## Discussion

In the investigation of social inference and human mate preference, a wide but inconsistent array of tools for computing scores of facial masculinity has been devised (see also [50]). Several of these approaches implicitly assumed that the individual expression of dimorphic shape features, which we refer to as *maleness*, resembles shape features perceived as masculine. We outlined a morphometric strategy for estimating separately the face shape patterns that underlie perceived masculinity and maleness by regressions of shape on rated masculinity and sex, respectively. Geometric morphometrics allows for the computation of shape scores for perceived masculinity and for maleness, as well as for the visualization of these shape patterns and the construction of faces with different degrees of masculinity or maleness.

When we applied these methods to a set of facial photographs, we found that shape features typically perceived as masculine are wide faces with a wide inter-orbital distance, a wide nose, thin lips, and a large and massive mandible. The individual scores for this combination of shape features—the masculinity shape scores—had a correlation with the actual masculinity ratings of 0.5, which clearly exceeds the correlations reported in Sanchez-Pages et al. [50] for a range of univariate and multivariate morphometric estimates of masculinity. In our data, the estimated pattern of face shape thus accounts for 25% of the variation in masculinity ratings. The remaining 75% of variation must be attributable to other factors, including variation in facial texture and hair color. Despite great efforts to photograph all individuals in a standardized way, variation in head posture probably contributes to the unexplained variation (however, it does not seem to have a systematic effect in our data because the shape patterns depicted by the deformation grids are more local and not related to head posture). Furthermore, it is known that the phase of menstrual cycle and other aspects affecting the psychological status influence the perception and preference of male face stimuli ([51], but see [8]). It has also been reported that facial asymmetry is related to measures of masculinity [52], and we find a correlation of 0.42 between rated masculinity and facial fluctuating asymmetry [46] in our data. The effect of fluctuating facial asymmetry on the rating is not accounted for by the regression approach and, hence, contributes to the unexplained variance.

The pattern of sexual dimorphism only partly resembled the shape features of perceived masculinity. The maleness shape scores, i.e., the individual scores along the sexual dimorphism vector, accounted for 7% of variation of the masculinity rating. This is slightly below the correlation of 0.33 (*R* ^2^ = 0.11) reported by Komori et al. [32]. When decomposing sexual dimorphism into an allometric and a non-allometric part, the relation to the masculinity rating was largely driven by the allometric part, despite similar variation of both parts in male faces. The pattern of allometry in face shape, mainly involving the lower face, was similar to the one found in Mitteroecker et al. [13]. Even though the faces were all scaled to the same size for the rating, face shape appears to contain cues to facial size, which we found to be correlated with rated masculinity.

The discriminant function was not successful in predicting rated masculinity, providing for less than 2% of explained variance. Furthermore, the discriminant function considerably depended on the number of selected principal components. The shape features combined by the discriminant function resembled neither the pattern of sexual dimorphism nor the pattern of perceived masculinity. Likewise, the Procrustes distance between male shapes and the female average, as suggested by Sanchez-Pages and Turiegano [43], accounted for only 3.6% of variation in the rating.

To conclude, proper quantification of the influence of biological factors such as size, hormones, immunocompetence, and body composition, on perceived masculinity and attractiveness via facial shape is a promising direction of research for understanding social behavior and the evolution of human mate choice. The distinction between the perceived masculinity of a face and its expression of sexually dimorphic shape characteristics (which we termed maleness) is crucial in this research agenda. Discriminant functions and Procrustes distances to the female mean shape are poor predictors of perceived masculinity. Still, our findings show that it is possible to estimate the shape pattern of sexual dimorphism in human faces, and we can compute scores that are maximally dimorphic (maleness shape scores) in order to assess individual variation of secondary facial sex characteristics. Furthermore, it is possible to compute the shape pattern that corresponds, on average, to high or low perceived masculinity ratings. The individual expressions of this average pattern (masculinity shape scores) provide the best predictor of rated masculinity. These are two principal approaches to the study of sexual dimorphism and perceived masculinity in the human face. It remains to be evaluated to which degree the relationship between face shape and perceived masculinity is indeed linear and which threshold levels exist for this relationship at extreme ranges of variation.
